# Tracking Treatment Response in Cardiac Light-Chain Amyloidosis With Native T1 Mapping

**DOI:** 10.1001/jamacardio.2023.2010

**Published:** 2023-07-19

**Authors:** Adam Ioannou, Rishi K. Patel, Ana Martinez-Naharro, Yousuf Razvi, Aldostefano Porcari, Muhammad U. Rauf, Roos E. Bolhuis, Jacob Fernando-Sayers, Ruta Virsinskaite, Francesco Bandera, Tushar Kotecha, Lucia Venneri, Daniel Knight, Charlotte Manisty, James Moon, Helen Lachmann, Carol Whelan, Peter Kellman, Philip N. Hawkins, Julian D. Gillmore, Ashutosh Wechalekar, Marianna Fontana

**Affiliations:** 1 National Amyloidosis Centre, Royal Free Campus, University College London, London, United Kingdom; 2 Center for Diagnosis and Treatment of Cardiomyopathies, Cardiovascular Department, Azienda Sanitaria Universitaria Giuliano-Isontina (ASUGI), University of Trieste, Trieste, Italy; 3 Cardiology University Department, IRCCS Policlinico San Donato, Milan, Italy; 4 St Bartholomew’s Hospital, London, United Kingdom; 5 National Heart, Lung and Blood Institute, National Institutes of Health, Bethesda, Maryland

## Abstract

**Importance:**

Cardiac magnetic resonance (CMR) imaging–derived extracellular volume (ECV) mapping, generated from precontrast and postcontrast T1, accurately determines treatment response in cardiac light-chain amyloidosis. Native T1 mapping, which can be derived without the need for contrast, has demonstrated accuracy in diagnosis and prognostication, but it is unclear whether serial native T1 measurements could also track the cardiac treatment response.

**Objective:**

To assess whether native T1 mapping can measure the cardiac treatment response and the association between changes in native T1 and prognosis.

**Design, Setting, and Participants:**

This single-center cohort study evaluated patients diagnosed with cardiac light-chain amyloidosis (January 2016 to December 2020) who underwent CMR scans at diagnosis and a repeat scan following chemotherapy. Analysis took place between January 2016 and October 2022.

**Main Outcomes and Measures:**

Comparison of biomarkers and cardiac imaging parameters between patients with a reduced, stable, or increased native T1 and association between changes in native T1 and mortality.

**Results:**

The study comprised 221 patients (mean [SD] age, 64.7 [10.6] years; 130 male [59%]). At 6 months, 183 patients (mean [SD] age, 64.8 [10.5] years; 110 male [60%]) underwent repeat CMR imaging. Reduced native T1 of 50 milliseconds or more occurred in 8 patients (4%), all of whom had a good hematological response; by contrast, an increased native T1 of 50 milliseconds or more occurred in 42 patients (23%), most of whom had a poor hematological response (27 [68%]). At 12 months, 160 patients (mean [SD] age, 63.8 [11.1] years; 94 male [59%]) had a repeat CMR scan. A reduced native T1 occurred in 24 patients (15%), all of whom had a good hematological response, and was associated with a reduction in N-terminal pro-brain natriuretic peptide (median [IQR], 2638 [913-5767] vs 423 [128-1777] ng/L; *P* < .001), maximal wall thickness (mean [SD], 14.8 [3.6] vs 13.6 [3.9] mm; *P* = .009), and E/e' (mean [SD], 14.9 [6.8] vs 12.0 [4.0]; *P* = .007), improved longitudinal strain (mean [SD], −14.8% [4.0%] vs −16.7% [4.0%]; *P* = .004), and reduction in both myocardial T2 (mean [SD], 52.3 [2.9] vs 49.4 [2.0] milliseconds; *P* < .001) and ECV (mean [SD], 0.47 [0.07] vs 0.42 [0.08]; *P* < .001). At 12 months, an increased native T1 occurred in 24 patients (15%), most of whom had a poor hematological response (17 [71%]), and was associated with an increased N-terminal pro-brain natriuretic peptide (median [IQR], 1622 [554-5487] vs 3150 [1161-8745] ng/L; *P* = .007), reduced left ventricular ejection fraction (mean [SD], 65.8% [11.4%] vs 61.5% [12.4%]; *P* = .009), and an increase in both myocardial T2 (mean [SD], 52.5 [2.7] vs 55.3 [4.2] milliseconds; *P* < .001) and ECV (mean [SD], 0.48 [0.09] vs 0.56 [0.09]; *P* < .001). Change in myocardial native T1 at 6 months was independently associated with mortality (hazard ratio, 2.41 [95% CI, 1.36-4.27]; *P* = .003).

**Conclusions and Relevance:**

Changes in native T1 in response to treatment, reflecting a composite of changes in T2 and ECV, are associated with in changes in traditional markers of cardiac response and associated with mortality. However, as a single-center study, these results require external validation in a larger cohort.

## Key Points

**Question:**

Can changes in myocardial native T1, derived without the need for contrast, track the treatment response in cardiac light-chain amyloidosis?

**Findings:**

In this cohort study, 221 patients had repeat cardiac magnetic resonance imaging 6 and/or 12 months after initiating chemotherapy. Changes in myocardial native T1 in response to treatment reflect a composite of changes in myocardial T2 and extracellular volume; changes in native T1 are also associated with in changes in traditional validated markers of the cardiac response and are independently associated with mortality.

**Meaning:**

Changes in myocardial native T1 may be used in the clinical setting to track treatment response in cardiac light-chain amyloidosis.

## Teaser

This cohort study assesses whether native T1 mapping can measure the cardiac light-chain amyloidosis treatment response and the association between changes in native T1 and prognosis.

## Introduction

Cardiac light-chain (AL) amyloidosis is caused by monoclonal immunoglobulin light-chains that misfold into pathogenic amyloid fibrils and deposit in the myocardial interstitial space.^1,2^ Cardiac magnetic resonance (CMR) imaging–derived extracellular volume (ECV) mapping is generated from precontrast and postcontrast T1 and requires gadolinium contrast to enable isolation of the extracellular signal with ECV measurements most likely quantifying amyloid accumulation and changes representing a measure of treatment response.^3,4,5^ Native T1, which can be derived without the need for contrast, provides a composite signal influenced by the intracellular and extracellular space. Although native T1 has demonstrated accuracy in diagnosis and prognostication, it is unclear whether serial measurements could track the cardiac response to treatment.^6,7,8^

The aims of this study were to assess the ability of CMR with native T1 mapping to measure the cardiac response to treatment and the association between changes in native T1 and prognosis.

## Methods

Study participants comprised individuals with cardiac AL amyloidosis diagnosed at the National Amyloidosis Centre (January 2016 to December 2020). Data from 176 patients had been included within a prior publication from our center, but the current study includes an updated analysis.^5^ Data on ethnicity were self-reported.

Ethical approval and CMR protocols are described in the eAppendix in Supplement 1. Patients were managed in accordance with the Declaration of Helsinki^9^ and provided written informed consent for analysis and publication of their data. Comparisons were made between baseline and follow-up CMR scans to calculate the absolute change in native T1. Patients were classified as having a reduced native T1 (native T1 reduction ≥50 milliseconds), a stable native T1 (change in native T1 <50 milliseconds), or an increased native T1 (native T1 increase ≥50 milliseconds) (eFigure 2 in Supplement 1).

Continuous variables are presented as mean (SD) or median (IQR), other than N-terminal pro-brain natriuretic peptide (NT-proBNP), which was natural log transformed for parametric testing. Continuous variables were analyzed using the independent *t* test or 1-way analysis of variance if the data were normally distributed or their nonparametric equivalents if data were not normally distributed. Categorical data are presented as absolute numbers and frequencies (%) and compared using the χ^2^ test. Correlation between parameters were assessed using Pearson *r* or Spearman ρ.

All mortality data were obtained via the UK Office of National Statistics, which is the national government registry for all deaths. Survival was evaluated with Cox proportional hazards regression analysis. Multivariable models were used to investigate the association between the change in native T1 or change in ECV with mortality. Akaike information criterion and Harrell C statistics were calculated for each model, and Harrell C statistics were compared using the Wald test. Kaplan-Meier curves were constructed, with statistical significance being assessed with a log-rank test. *P* values were 2-sided, and statistical significance was defined as *P* < .05, except for paired tests where significance was defined as *P* < .01. Analysis took place between January 2016 and October 2022.

## Results

The study comprised 221 patients with a mean (SD) age of 64.7 (10.6) years, 130 (58.8%) were male, and the mean (SD) body surface area was 1.88 (0.25) m^2^. Overall, 6 patients (2.7%) were Asian, 14 (6.3%) were Black, and 201 (91.0%) were White. The median (interquartile range) NT-proBNP was 2443 (926-5230) ng/L, median (IQR) troponin T was 0.052 (0.028-0.100) ng/mL (to convert to micrograms per liter, multiply by 1), and 102 (46.2%) had Mayo stage IIIa biomarkers at diagnosis (eTables 1 and 2 and eFigure 1 in Supplement 1). All patients underwent a CMR at diagnosis, 183 patients (mean [SD] age, 64.8 [10.5] years; 110 [60%] male) had repeat CMR imaging 6 months after commencing chemotherapy, and 160 patients (mean [SD] age, 63.8 [11.1] years; 94 [59%] male) had repeat CMR imaging 12 months after commencing chemotherapy. The 12-month follow-up cohort comprised 145 patients who also underwent CMR imaging at 6 months and 38 patients whose first follow-up CMR imaging was at 12 months.

### CMR Findings 6 Months Postchemotherapy

A reduced native T1 was detectible in 8 patients (4.4%), 130 patients (71.0%) had a stable native T1, and 45 (24.6%) had an increased native T1. All 8 patients with a reduced native T1 achieved a good hematological response (complete response, 7; very good partial response, 1), whereas the majority of patients with an increased native T1 had a poor hematological response (partial response or no response, 27 [60.0%]) (eTable 3 in Supplement 1).

Patients with a reduced native T1 had a significant reduction in T2 (mean [SD], 52.0 [2.4] vs 48.9 [2.0] milliseconds; *P* = .002) and ECV (mean [SD], 0.46 [0.05] vs 0.40 [0.06]; *P* = .009). Those who received contrast demonstrated ECV regression (4 [57.1%]) or a stable ECV (3 [42.9%]). All patients with a reduced native T1, but a stable ECV, demonstrated a reduction in T2 of 3 milliseconds or more.

Patients with an increased native T1 had a significant increase in T2 (mean [SD], 52.2 [2.7] vs 55.5 [4.0] milliseconds; *P* < .001) and ECV (mean [SD], 0.49 [0.08] vs 0.57 [0.09]; *P* < .001). The majority who received contrast demonstrated ECV progression (28 [71.8%]), while the remaining patients had a stable ECV (11 [28.2%]). Subgroup analysis of patients with an increased native T1, but stable ECV, demonstrated these patients had a significant increase in T2 (mean [SD], 51.9 [3.8] vs 56.6 [4.0] milliseconds; *P* < .001) and no significant change in ECV (mean [SD], 0.53 [0.09] vs 0.54 [0.09]; *P* = .04) (eTable 4 in Supplement 1).

### CMR Findings 12 Months Postchemotherapy

A reduced native T1 was detectible in 24 patients (15.0%), 112 patients (70.0%) had a stable native T1, and 24 (15.0%) had an increased native T1. All 24 patients with a reduced native T1 achieved a good hematological response (complete response, 18 [75.0%]; very good partial response, 6 [25.0%]), whereas the majority of patients with an increased native T1 had a poor hematological response (partial response or no response, 17 [70.8%]) (eTable 5 in Supplement 1).

Patients with a reduced native T1 had a significant reduction in NT-proBNP (median [IQR], 2638 [913-5767] vs 423 [128-1777] ng/L; *P* < .001), maximal wall thickness (mean [SD], 14.9 [6.8] vs 13.6 [3.9] mm; *P* = .009), and E/e' (mean [SD], 14.9 [6.8] vs 12.0 [4.0]; *P* = .007) and improved longitudinal strain (mean [SD], −14.8% [4.0%] vs −16.7% [4.0%]; *P* = .004). There was also a significant reduction in both T2 (mean [SD], 52.3 [2.9] vs 49.4 [2.0] milliseconds; *P* < .001) and ECV (mean [SD], 0.47 [0.07] vs 0.42 [0.08]; *P* < .001). The majority who received gadolinium contrast had ECV regression (18 [78.3%]), while the remaining patients had a stable ECV (5 [21.7%]). All patients with a reduced native T1 but a stable ECV demonstrated a reduction in T2 of 3 milliseconds or more.

Patients with an increased native T1 had a significant increase in NT-proBNP (median [IQR], 1622 [554-5487] vs 3150 [1161-8745] ng/L; *P* = .007) and reduction in left ventricular ejection fraction (mean [SD], 65.8% [11.4%] vs 61.5% [12.4%]; *P* = .009). There was also a significant increase in both T2 (mean [SD], 52.5 [2.7] vs 55.3 [4.2] milliseconds; *P* < .001) and ECV (mean [SD], 0.48 [0.09] vs 0.56 [0.09]; *P* < .001). The majority who received gadolinium contrast demonstrated ECV progression (22 [95.6%]), while the remaining patient had a stable ECV but an increase in T2 of 3 milliseconds or more (Figure A and B; eTable 6 and eFigures 3 and 4 in Supplement 1).

**Figure.**
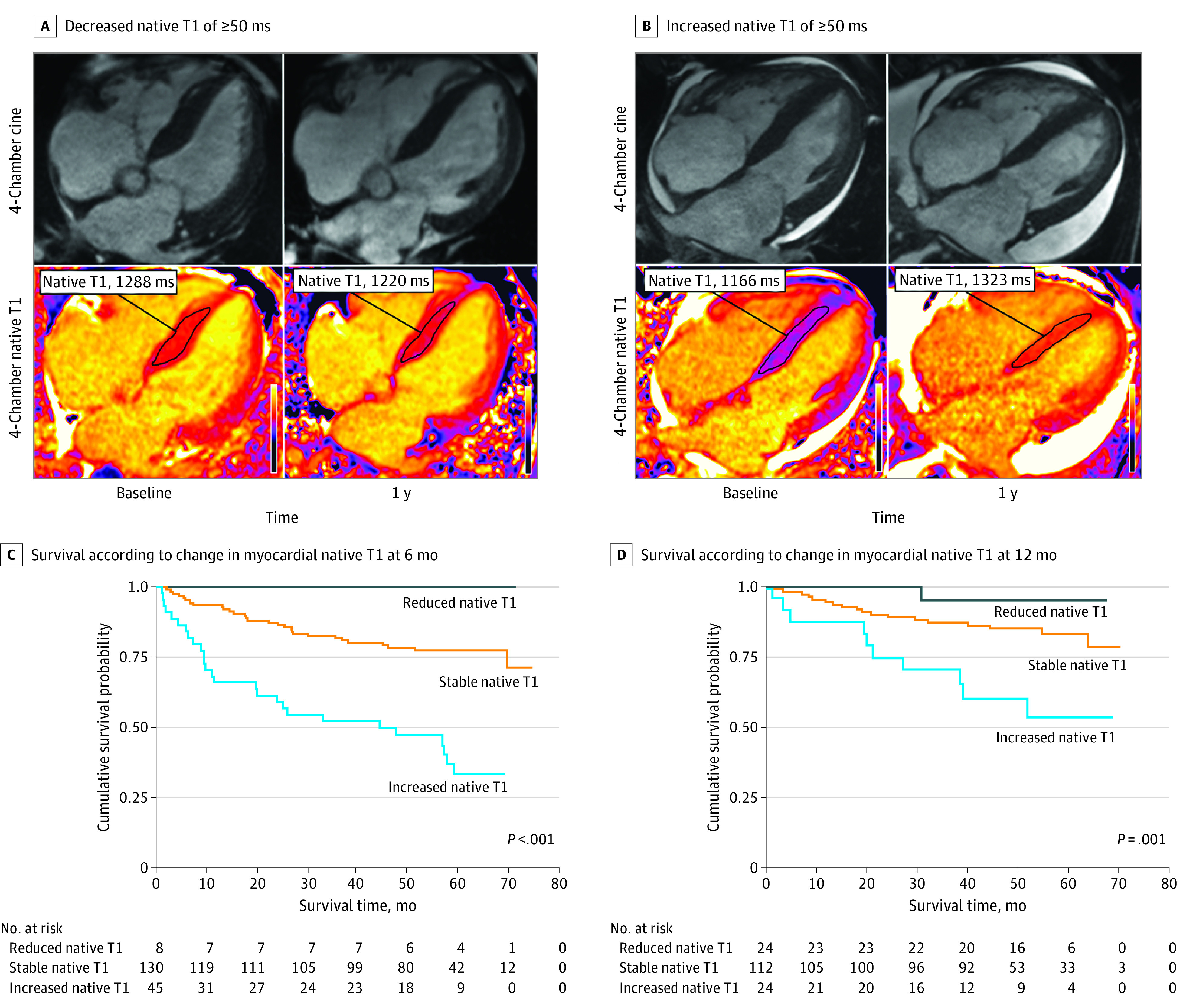
Changes in Native T1 and Associated Survival Cardiac magnetic resonance images demonstrating reduced myocardial native T1 of 50 milliseconds or more (A) and increased myocardial native T1 of 50 milliseconds or more (B). Kaplan-Meier curve demonstrating the association between change in myocardial native T1 and survival 6 months after initiating chemotherapy (C). Kaplan-Meier curve demonstrating the association between change in myocardial native T1 and survival 12 months after initiating chemotherapy (D).

Change in native T1 and change in ECV had a strong positive correlation (*R* = 0.699; *P* < .001), while the percentage change in NT-proBNP had a weak positive correlation with the change in native T1 (*R* = 0.443; *P* < .001) and a moderate positive correlation with the change in ECV (*R* = 0.525; *P* < .001).

### Survival

At median (IQR) follow-up of 60 (37-71) months from the time of diagnosis, 62 patients (28.1%) had died. Patients with an increased native T1 at 6 or 12 months had a worse prognosis (Figure C and D). Two multivariable models adjusting for hematological and NT-proBNP response demonstrated that change in native T1 and ECV at 6 months remained independently associated with mortality (increased native T1: hazard ratio, 2.41 [95% CI, 1.36-4.27]; *P* = .003; ECV progression: hazard ratio, 4.67 [95% CI, 2.41-9.06; *P* < .001). The multivariable model containing change in ECV had a lower Akaike information criterion than the model containing native T1, but the Harrell C statistics of both models were not significantly different (eTable 7 in Supplement 1).

## Discussion

In this prospective study, we demonstrate that native T1 can track the treatment response in cardiac AL amyloidosis. Change in native T1 reflects the composite change of ECV and T2 and is independently associated with mortality.

Cardiac response is commonly measured using NT-proBNP, with a reduction thought to reflect diminished cardiotoxicity and being associated with favorable outcomes.^10^ Native T1 complements conventional methods of monitoring treatment response, with change in native T1 being independently associated with mortality and hence redefining the treatment response beyond traditional markers.

Native T1 is closely related to ECV, with ECV maps being generated from precontrast and postcontrast T1.^11^ As expected, change in ECV correlated with change in native T1, but change in ECV demonstrated a stronger correlation with change in NT-proBNP and the multivariable model containing change in ECV had a lower Akaike information criterion than the model containing native T1. Despite this, the multivariable model containing ECV had a similar Harrell C statistic to the model containing native T1, confirming the role of native T1 as an effective marker of treatment response.

These differences may represent the different biological data being measured. Amyloid fibril accumulation causes ECV expansion as well as myocardial damage and edema secondary to light-chain toxicity and rapid amyloid accumulation reflected by increased T2. ECV measurements most likely quantify the degree of amyloid deposition, whereas native T1 provides a composite signal influenced by amyloid accumulation (ECV) and myocardial edema (T2) (eFigure 5 in Supplement 1).^5,12,13^

Serial native T1 measurements represent an important addition to the standard armamentarium of amyloid assessment. Changes in ECV are able to track the cardiac treatment response, but ECV mapping requires gadolinium contrast administration.^5^ Concerns have been raised regarding the risk of contrast administration in the setting of chronic kidney disease and also regarding accumulation of gadolinium deposits in the brain. These concerns resulted in the removal of linear agents for most indications, macrocylic agents being used at the lowest possible dose, and only when noncontrast scans are not suitable. Serial native T1 measurements are able to track the cardiac treatment response while simultaneously avoiding contrast exposure. Use of native T1 without the need for contrast also reduces acquisition time by up to two-thirds, with noncontrast studies that include native T1 mapping, volume, and functional assessments being performed in less than 15 minutes. This additional advantage has implications on diagnostic workflow, with reductions in scan time resulting in improvements in efficiency and a greater capacity for more CMR scans to take place.^7,8^

### Limitations

Serial CMR scans were only available for patients with follow-up imaging, which invariably introduced survival bias. Rapid amyloid accumulation may have resulted in mortality before the 6-month interval CMR scan could take place. This is a single-center study and therefore requires validation in a larger cohort of patients.

## Conclusions

In summary, changes in native T1 in response to treatment are reflected in changes in the traditional markers of the cardiac response; importantly, changes in native T1 are independently associated with mortality. However, this is a single-center study, and therefore these results require external validation in a larger cohort of patients.

## Notes

Supplement 1. eAppendix. eTable 1. International consensus criteria for hematological response and N-terminal pro-brain natriuretic peptide (NT-proBNP) eTable 2. Demographics, serum biomarker, echocardiographic and cardiac magnetic resonance findings at diagnosis in patients with cardiac AL amyloidosis according to changes in native-T1 6-months after the initiation of chemotherapy eTable 3. Baseline demographics, serum biomarker, echocardiographic and cardiac magnetic resonance findings at diagnosis in patients with cardiac AL amyloidosis according to changes in native-T1 6-months after the initiation of chemotherapy eTable 4. Changes in serum biomarkers, echocardiographic and cardiac magnetic resonance findings in patients with cardiac AL amyloidosis according to changes in native-T1 6-months after the initiation of chemotherapy eTable 5. Baseline demographics, serum biomarker, echocardiographic and cardiac magnetic resonance findings at diagnosis in patients with cardiac AL amyloidosis according to changes in native-T1 12-months after the initiation of chemotherapy eTable 6. Changes in serum biomarkers, echocardiographic and cardiac magnetic resonance findings in patients with cardiac AL amyloidosis according to changes in native-T1 12-months after the initiation of chemotherapy eTable 7. Univariable and multivariable analysis of mortality risk 6-months after the initiation of chemotherapy eFigure 1. Flow chart demonstrating which patients had follow up imaging eFigure 2. Bland-Altman plot of the myocardial native-T1 measured in patients with end-stage renal failure before and immediately after haemodialysis eFigure 3. Follow chart summarizing the longitudinal data for patients who underwent cardiac magnetic resonance (CMR) scans at all three timepoints (baseline, 6-months and 12-months) eFigure 4. Illustration of the changes observed in serum biomarkers, echocardiographic and cardiac magnetic resonance parameters following changes in myocardial native-T1 eFigure 5. Illustration of how changes in myocardial T2 and ECV influence changes in myocardial native-T1

Supplement 2. Data sharing statement
