# Supplementary material for: Tracking Treatment Response in Cardiac Light-Chain Amyloidosis With Native T1 Mapping
Source: JAMA Cardiol. 2023 Jul 19;8(9):848–52. doi: 10.1001/jamacardio.2023.2010 (PMC10357357; doi:10.1001/jamacardio.2023.2010)
Supplement: Supplement 1. — eAppendix. eTable 1. International consensus criteria for hematological response and N-terminal pro-brain natriuretic peptide (NT-proBNP) eTable 2. Demographics, serum biomarker, echocardiographic and cardiac magnetic resonance findings at diagnosis in patients with cardiac AL amyloidosis according to changes in native-T1 6-months after the initiation of chemotherapy eTable 3. Baseline demographics, serum biomarker, echocardiographic and cardiac magnetic resonance findings at diagnosis in patients with cardiac AL amyloidosis according to changes in native-T1 6-months after the initiation of chemotherapy eTable 4. Changes in serum biomarkers, echocardiographic and cardiac magnetic resonance findings in patients with cardiac AL amyloidosis according to changes in native-T1 6-months after the initiation of chemotherapy eTable 5. Baseline demographics, serum biomarker, echocardiographic and cardiac magnetic resonance findings at diagnosis in patients with cardiac AL amyloidosis according to changes in native-T1 12-months after the initiation of chemotherapy eTable 6. Changes in serum biomarkers, echocardiographic and cardiac magnetic resonance findings in patients with cardiac AL amyloidosis according to changes in native-T1 12-months after the initiation of chemotherapy eTable 7. Univariable and multivariable analysis of mortality risk 6-months after the initiation of chemotherapy eFigure 1. Flow chart demonstrating which patients had follow up imaging eFigure 2. Bland-Altman plot of the myocardial native-T1 measured in patients with end-stage renal failure before and immediately after haemodialysis eFigure 3. Follow chart summarizing the longitudinal data for patients who underwent cardiac magnetic resonance (CMR) scans at all three timepoints (baseline, 6-months and 12-months) eFigure 4. Illustration of the changes observed in serum biomarkers, echocardiographic and cardiac magnetic resonance parameters following changes in myocardial native-T1 eFigure 5. Illustratio [file jamacardiol-e232010-s001.pdf]

Supplemental Online Content

Ioannou A, Patel RK, Martinez-Naharro A, et al. Tracking treatment response in cardiac light-chain amyloidosis with native T1 mapping. *JAMA Cardiol*. Published online July 19, 2023. doi:10.1001/jamacardio.2023.2010

eAppendix.
eTable 1. International consensus criteria for haematological response and N-terminal pro-brain natriuretic peptide (NT-proBNP)
eTable 2. Demographics, serum biomarker, echocardiographic and cardiac magnetic resonance findings at diagnosis in patients with cardiac AL amyloidosis according to changes in native-T1 6-months after the initiation of chemotherapy
eTable 3. Baseline demographics, serum biomarker, echocardiographic and cardiac magnetic resonance findings at diagnosis in patients with cardiac AL amyloidosis according to changes in native-T1 6-months after the initiation of chemotherapy
eTable 4. Changes in serum biomarkers, echocardiographic and cardiac magnetic resonance findings in patients with cardiac AL amyloidosis according to changes in native-T1 6-months after the initiation of chemotherapy
eTable 5. Baseline demographics, serum biomarker, echocardiographic and cardiac magnetic resonance findings at diagnosis in patients with cardiac AL amyloidosis according to changes in native-T1 12-months after the initiation of chemotherapy
eTable 6. Changes in serum biomarkers, echocardiographic and cardiac magnetic resonance findings in patients with cardiac AL amyloidosis according to changes in native-T1 12-months after the initiation of chemotherapy
eTable 7. Univariable and multivariable analysis of mortality risk 6-months after the initiation of chemotherapy
eFigure 1. Flow chart demonstrating which patients had follow up imaging
eFigure 2. Bland-Altman plot of the myocardial native-T1 measured in patients with end-stage renal failure before and immediately after haemodialysis
eFigure 3. Follow chart summarizing the longitudinal data for patients who underwent cardiac magnetic resonance (CMR) scans at all three timepoints (baseline, 6-months and 12-months)
eFigure 4. Illustration of the changes observed in serum biomarkers, echocardiographic and cardiac magnetic resonance parameters following changes in myocardial native-T1
eFigure 5. Illustration of how changes in myocardial T2 and ECV influence changes in myocardial native-T1

This supplemental material has been provided by the authors to give readers additional information about their work.

© 2023 Ioannou A et al. *JAMA Cardiololgy.*

© 2023 Ioannou A et al. *JAMA Cardiololgy*.

eAppendix.

Ethical approval

Patients were managed in accordance with the Declaration of Helsinki and provided written informed consent for analysis and publication of their data (REC reference: 09/H0715/58). A separate ethical approval was obtained for recruitment of patients who did not have systemic AL-amyloidosis, but had a diagnosis of end-stage renal failure (ESRF) and were established on haemodialysis. These patients underwent non-contrast CMR before and immediately after haemodialysis, and their images were analysed in order to establish a cut off for the change in native-T1 that would exceed any change attributable to fluid shift, or measurement error (REC reference: 07/H0715/101).

© 2023 Ioannou A et al. *JAMA Cardiololgy.*

Cardiac Magnetic Resonance Imaging

All subjects underwent CMR on a 1.5-T clinical scanner with localizers and cine imaging with steady state free precession sequence (SSFP). Native-T1 mapping was acquired using the modified look-locker inversion recovery sequence. For patients who received gadolinium contrast, late gadolinium enhancement (LGE) imaging was acquired with both magnitude inversion recovery and phase-sensitive inversion recovery sequence reconstructions with SSFP read-outs. After a bolus of gadoterate meglumine and LGE imaging, T1 mapping was repeated 15-minutes post-contrast using the same slice locations with the modified look-locker inversion recovery sequence, to produce automated inline ECV mapping reconstruction. T1-mapping protocols used 5s(3s)3s and 4s(1s)3s(1s)2s sampling, pre- and post-contrast, respectively. Patients with an estimated glomerular filtration rate (eGFR) <30ml/min/1.73m^2 who were not consented for the risks associated with gadolinium did not receive contrast and had a non-contrast CMR with pre contrast native-T1 mapping. Patients who developed renal impairment (eGFR <30ml/min/1.73m^2) who were not consented for the risks associated with gadolinium did not receive contrast and had a non-contrast CMR, even if contrast was administered during their baseline scan.

CMR analysis

All CMR image analysis was performed offline using Osirix MD 9.0 (Bernex, Switzerland) and reporting clinicians were blinded to all other clinical data. Native-T1, T2 and ECV measurements were obtained by drawing a single region of interest in the basal to mid septum of the appropriate 4-chamber map. Follow-up CMR scans that took place 6 and/or 12-months after commencing chemotherapy, were compared with baseline scans to determine the change in native-T1. Only those patients who had a CMR scan at each timepoint were included in the analysis pertaining to that timepoint.

© 2023 Ioannou A et al. *JAMA Cardiololgy.*

Native-T1 cut off

We included 25 patients who did not have systemic AL-amyloidosis, but had ESRF requiring haemodialysis (mean age:63.9±10.6years, male:68.0%) and who had repeated CMR scans before and immediately after haemodialysis. When compared to the 221 patients (mean age:64.7±10.6years, male:58.8%) diagnosed with cardiac AL-amyloidosis there was no significant difference in age (P=0.853) or proportion of males (P=0.375). The cut-off for change in myocardial native-T1 in response to treatment was determined following the analysis of the CMR scans that took place before and immediately after hemodialysis in the cohort with ESRF. Native-T1 measurements were obtained by drawing a single region of interest in in the basal to mid septum of the appropriate 4-chamber map. Haemodialysis resulted in a bias of -5.84ms (95%CI: -39.93 to 28.25) (Supplementary Figure S2). Therefore, an absolute change in native-T1 of ≥50ms, was far greater than any change attributable to fluid shift, or measurement error, and was considered a significant change in native-T1. Following this analysis, patients with cardiac AL-amyloidosis were classified as having a native-T1 reduction (native-T1 reduction ≥50ms), a stable native-T1 (change in native-T1 <50ms) or a native-T1 increase (native-T1 increase ≥50ms). A significant change in the myocardial ECV was considered as previously described as an absolute change in ECV of 0.05.

© 2023 Ioannou A et al. *JAMA Cardiololgy.*

Subgroup analysis of patients with 6-month and 12-month CMR scans

We identified 122 patients who had follow up CMR scans at both 6 and 12-months. Of this subgroup, there were 4 patients who demonstrated a native-T1 reduction ≥50ms at 6-months, and all 4 patients had a native-T1 reduction of ≥50ms at 12-months. At 12-months, there were an additional 17 patients who had a stable native-T1 at 6-months and demonstrated a native-T1 reduction of ≥50ms at 12-months, all of whom demonstrated a sustained good haematological response between the two scans.

At 6-months, 24 patients demonstrated an increase in native-T1 ≥50ms, 14 of also whom had an increase in native-T1 ≥50ms at 12-months, and 9 patients who no longer had an increased native-T1 ≥50ms at 12-months. The 9 patients who had an increase in native-T1 at 6-months and a stable native-T1 at 12-months (compared with their baseline CMR scan) all demonstrated a sustained good haematological response between the two scans (Supplementary Figure S3 and S4).

© 2023 Ioannou A et al. *JAMA Cardiololgy.*

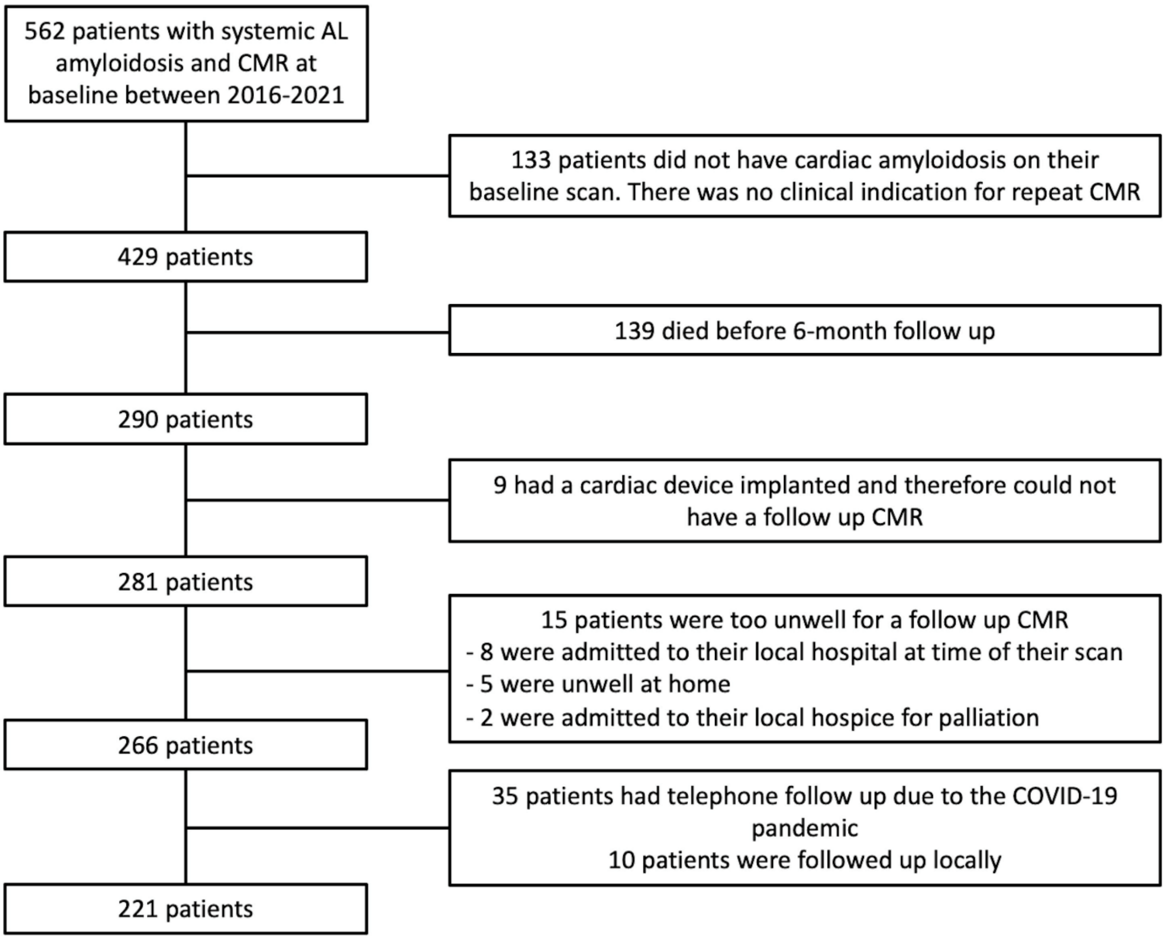

eFigure 1. Flow chart demonstrating which patients had follow up imaging.

© 2023 Ioannou A et al. *JAMA Cardiololgy.*

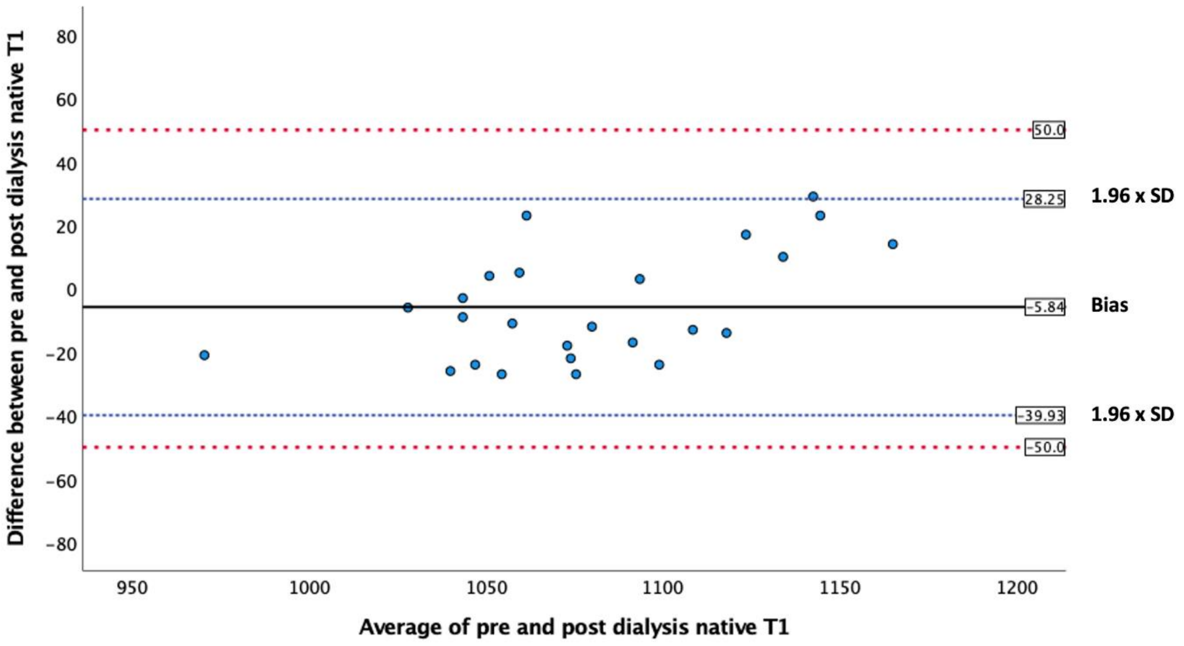

eFigure 2. Bland-Altman plot of the myocardial native-T1 measured in patients with end-stage renal failure before and immediately after haemodialysis.

© 2023 Ioannou A et al. *JAMA Cardiololgy.*

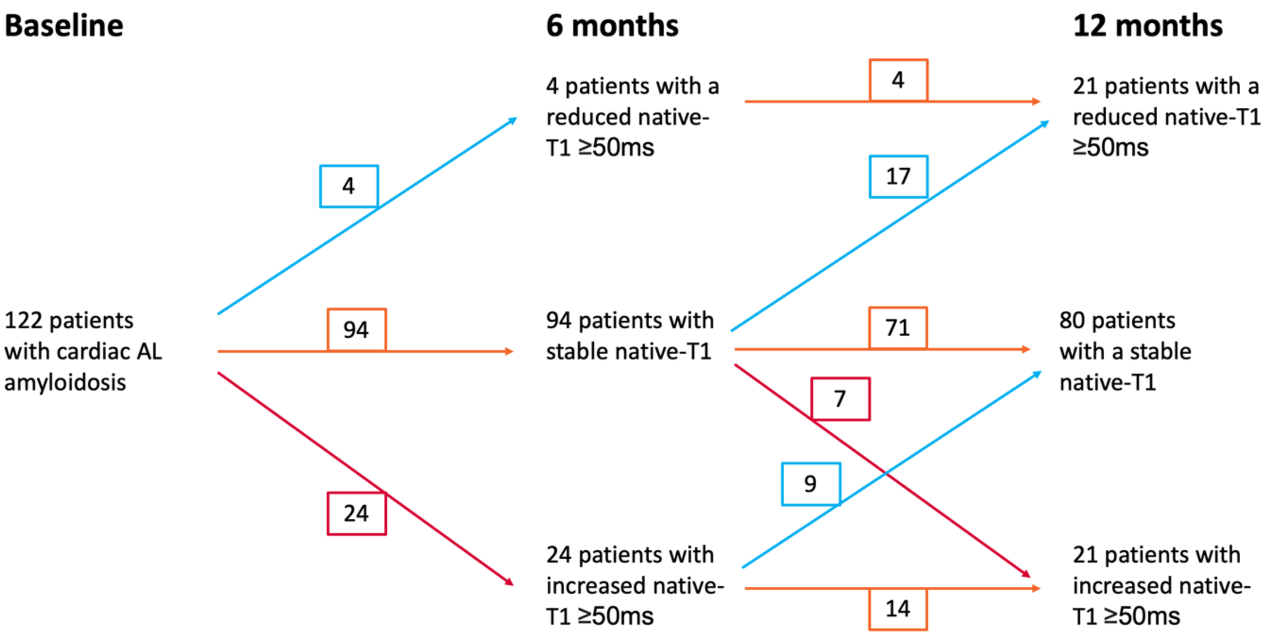

eFigure 3. Follow chart summarizing the longitudinal data for patients who underwent cardiac magnetic resonance (CMR) scans at all three timepoints (baseline, 6-months and 12-months).

© 2023 Ioannou A et al. *JAMA Cardiololgy.*

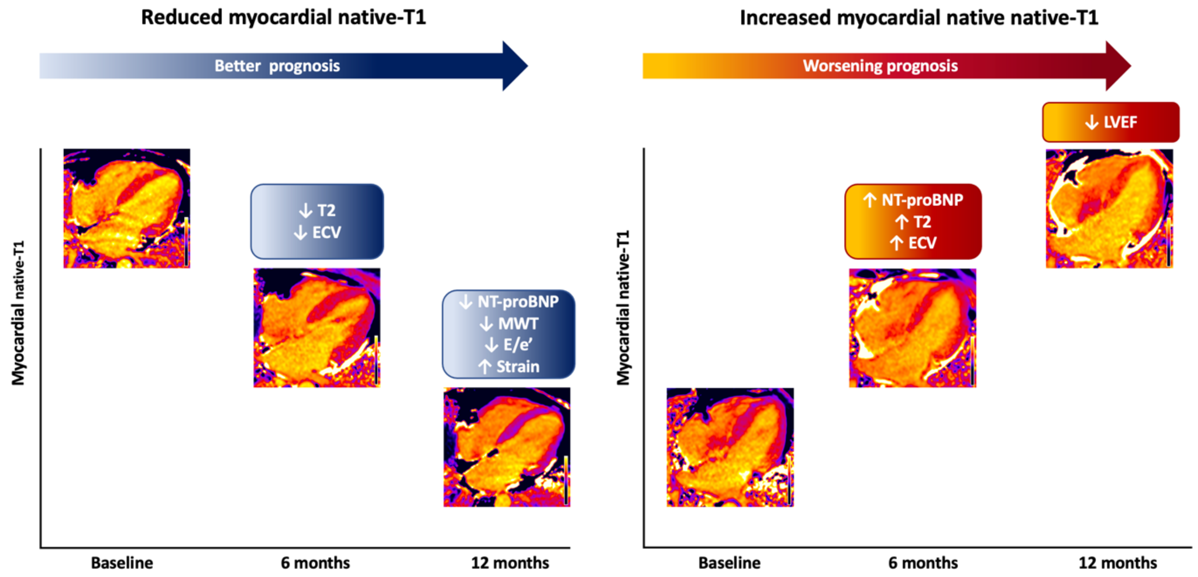

eFigure 4. Illustration of the changes observed in serum biomarkers, echocardiographic and cardiac magnetic resonance parameters following changes in myocardial native-T1.

© 2023 Ioannou A et al. *JAMA Cardiololgy.*

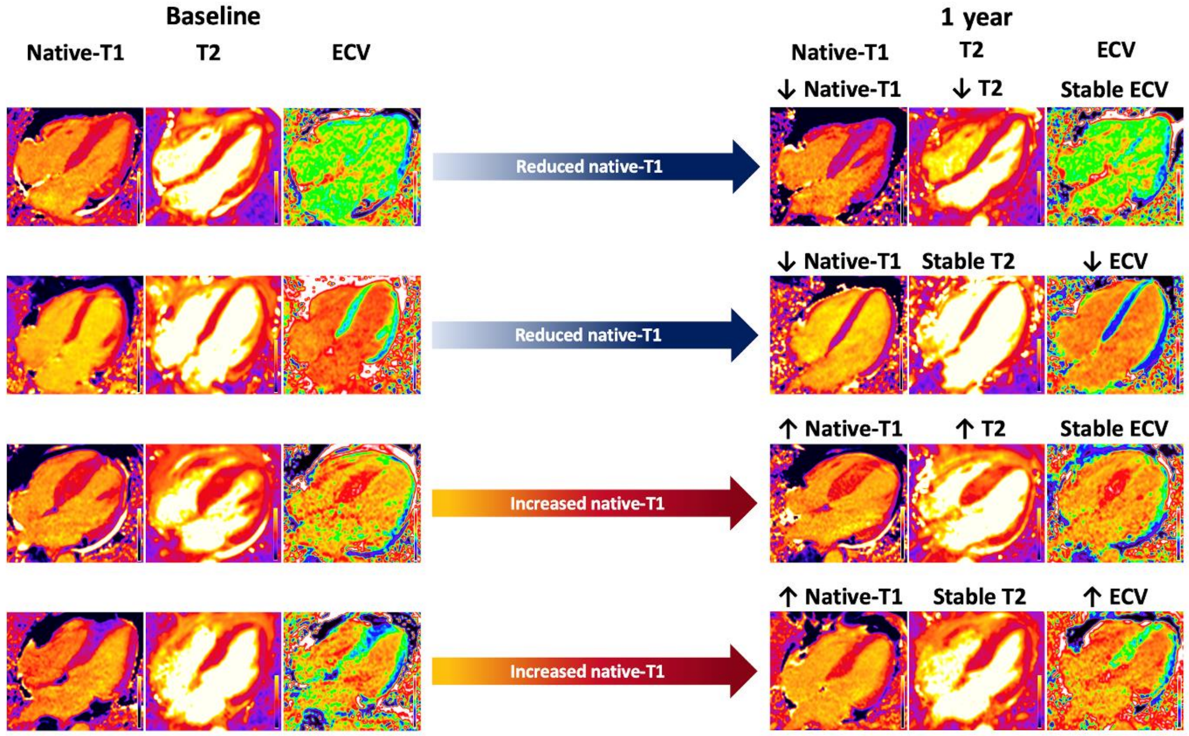

eFigure 5. Illustration of how changes in myocardial T2 and ECV influence changes in myocardial native-T1.

Haematological response	
Complete response (CR)	Normal free light-chains (FLC) levels, normal kappa/lambda ratio and negative serum immunofixation
Very good partial response (VGPR)	Reduction in dFLC [difference in concentration between aberrant and uninvolved class of FLC] to <40mg/L
Partial response (PR)	PR: >50% reduction in dFLC
No response (NR)	≤50% reduction in dFLC
**NT-proBNP response**	
NT-proBNP improvement	Reduction of >30% and >300ng/L
Stable NT-proBNP	Change of <30% or <300ng/L
NT-proBNP worsening	Increase of >30% and >300ng/L

eTable 1. International consensus criteria for haematological response and N-terminal pro-brain natriuretic peptide (NT-proBNP).

© 2023 Ioannou A et al. *JAMA Cardiololgy.*

Baseline characteristics (n = 221)	
**Demographics**	
Age (years)	64.7 ± 10.6
Sex (male)	130 (58.8%)
Body surface area (m^2)	1.88 ± 0.25
**Serum biomarkers**	
NT-proBNP (ng/L)	2443 (926 – 5230)
Troponin (ng/L)	52 (28 – 100)
dFLC (mg/L)	201 (71 – 427)
**Mayo stage**	
1	18 (8.1%)
2	77 (34.8%)
3a	102 (46.2%)
3b	21 (9.5%)
Missing	3
**Echocardiographic parameters**	
IVSd (mm)	14.1 ± 2.5
RWT	0.68 ± 0.17
E/e’	15.8 ± 6.8
LS (%)	-13.8 ± 4.9
**Cardiac magnetic resonance parameters**	
MWT (mm)	15.7 ± 3.9
LV mass indexed (g/m^2)	97.6 ± 34.0
LVEF (%)	64.3 ± 10.7
MAPSE (mm)	8.4 ± 3.2
TAPSE (mm)	16.1 ± 5.8
LA area (cm^2)	26.6 ± 7.3
RA area (cm^2)	22.5 ± 6.4
Native T1 (ms)	1165.7 ± 57.6
T2 (ms)	52.0 ± 2.9
ECV*	0.47 ± 0.08

eTable 2. Demographics, serum biomarker, echocardiographic and cardiac magnetic resonance findings at diagnosis in patients with cardiac AL amyloidosis according to changes in native-T1 6-months after the initiation of chemotherapy. *ECV measurements were available for 218 patients who received gadolinium contrast during their baseline scan.

NT-proBNP = N-terminal pro-brain natriuretic peptide, IVSd = Interventricular septal diameter in diastole, RWT = Relative wall thickness, LS = Longitudinal strain, MWT = Maximal wall thickness, LV = Left ventricular, LVEF = Left ventricular ejection fraction, MAPSE = Mitral annular plane systolic excursion, TAPSE = Tricuspid annular plane systolic excursion, LA = Left atrial, RA = Right atrial, ECV = Extracellular volume.

© 2023 Ioannou A et al. *JAMA Cardiololgy.*

Baseline characteristics				
	**Reduced native-T1 (n = 8, 4.4%)**	**Stable native-T1 (n = 130, 71.0%)**	**Increased native-T1 (n = 45, 24.6%)**	**P-value**
**Demographics**				
Age (years)	64.76±14.83	65.38±10.78	63.14±8.63	0.286
Sex (male)	3 (37.5%)	81 (62.3%)	26 (57.8%)	0.355
**Serum biomarkers**				
NT-proBNP (ng/L)	3896 (1326-8485)	2674 (941-5158)	1989 (670-5792)	0.421
Troponin (ng/L)	42 (34-74)	54 (29-106)	54 (26-130)	0.765
**Echocardiographic parameters**				
IVSd (mm)	12.63±2.62	14.16±2.43	14.42±2.82	0.267
RWT	0.60±0.12	0.69±0.16	0.68±0.16	0.224
E/e’	14.56±4.04	15.94±6.94	15.69±7.34	0.947
LS (%)	-14.14±4.61	-14.05±4.78	-13.92±5.64	0.986
**Cardiac magnetic resonance parameters**				
MWT (mm)	14.25±3.81	15.82±4.16	15.98±4.10	0.507
LV mass indexed (g/m^2)	80.00±23.60	98.57±34.35	102.60±39.71	0.268
LVEF (%)	63.50±14.85	64.61±9.69	63.62±12.43	0.922
MAPSE (mm)	6.83±1.94	8.40±3.08	8.71±3.29	0.462
TAPSE (mm)	15.00±4.84	16.31±5.31	16.36±7.02	0.805
LA area (cm^2)	23.13±6.64	27.12±7.12	27.91±7.61	0.352
RA area (cm^2)	20.00±6.74	22.78±6.36	23.76±6.02	0.248
Native T1 (ms)	1162.25±32.63	1168.19±61.71	1171.58±45.74	0.894
T2 (ms)	52.00±2.39	52.08±3.04	52.26±2.71	0.627
ECV*	0.45±0.05	0.47±0.08	0.49±0.08	0.290

eTable 3. Baseline demographics, serum biomarker, echocardiographic and cardiac magnetic resonance findings at diagnosis in patients with cardiac AL amyloidosis according to changes in native-T1 6-months after the initiation of chemotherapy. Reduced native-T1 was defined as a native-T1 reduction ≥50ms, stable native-T1 was defined as a change in native-T1 <50ms, and increased native-T1 was defined as a native-T1 reduction ≥50ms. *ECV measurements were available for the 164 patients who received gadolinium contrast during both their baseline and 6-month follow up CMR scans.

P-values for pairwise comparison: α = Regression vs Stable, β = Regression vs Progression. NT-proBNP = N-terminal pro-brain natriuretic peptide, IVSd = Interventricular septal diameter in diastole, RWT = Relative wall thickness, LS = Longitudinal strain, MWT = Maximal wall thickness, LV = Left ventricular, LVEF = Left ventricular ejection fraction, MAPSE = Mitral annular plane systolic excursion, TAPSE = Tricuspid annular plane systolic excursion, LA = Left atrial, RA = Right atrial, ECV = Extracellular volume.

© 2023 Ioannou A et al. *JAMA Cardiololgy.*

	Reduced native-T1 (n=8, 4.4%)			Stable native-T1 (n=130, 71.0%)			Increased native-T1 (n=45, 24.6%)		
	**Baseline**	**6 months**	**P-value**	**Baseline**	**6 months**	**P-value**	**Baseline**	**6 months**	**P-value**
**Serum biomarkers**									
Haematological response	CR = 7 (87.5%)<br>VGPR = 1 (12.5%)<br>PR = 0 (0.0%)<br>NR = 0 (0.0%)			CR = 63 (48.5%)<br>VGPR = 32 (24.6%)<br>PR = 25 (19.2%)<br>NR = 10 (7.7%)			CR = 9 (20.0%)<br>VGPR = 9 (20.0%)<br>PR = 16 (35.6%)<br>NR = 11 (24.4%)		
NT-proBNP (ng/L)	3772 (964-9210)	1146 (340-3037)	0.076	2817 (937-5275)	2295 (882-5010)	0.596	2349 (710-5802)	3136 (1685-10979)	0.002
NT-proBNP response	Improvement = 4 (50.0%)<br>Stable = 2 (25.0%)<br>Worse = 1 (12.5%)<br>Missing data = 1 (12.5%)			Improvement = 31 (23.8%)<br>Stable = 49 (37.7%)<br>Worse = 42 (32.3%)<br>Missing data = 8 (6.2%)			Improvement = 11 (24.4%)<br>Stable = 8 (17.8%)<br>Worse = 24 (53.3%)<br>Missing data = 2 (4.4%)		
**Echocardiographic parameters**									
IVSd (mm)	12.17±2.23	12.17±2.32	0.999	14.18±2.45	14.24±2.44	0.338	14.66±3.03	14.88±2.97	0.070
RWT	0.57±0.06	0.54±0.08	0.135	0.69±0.16	0.07±0.14	0.080	0.69±0.17	0.71±0.17	0.474
E/e’	15.25±4.37	12.30±5.36	0.237	15.67±6.94	15.39±6.11	0.590	16.05±7.67	16.61±7.84	0.455
LS (%)	-13.74±4.75	-14.64±4.20	0.200	-14.33±4.81	-13.89±4.62	0.167	-13.48±5.57	-12.98±4.07	0.375
**Cardiac magnetic resonance parameters**									
MWT (mm)	14.25±3.81	12.63±3.11	0.195	15.86±4.16	15.62±3.88	0.230	16.09±4.07	16.11±3.65	0.947
LV mass indexed (g/m^2)	80.00±23.60	74.38±17.12	0.253	98.57±34.35	97.42±34.23	0.515	102.60±39.71	101.47±35.96	0.638
LVEF (%)	63.00±14.85	64.38±14.15	0.742	64.61±9.69	63.72±10.45	0.204	63.62±12.45	60.63±13.31	0.012
MAPSE (mm)	6.83±1.94	7.00±2.90	0.867	8.49±3.05	8.30±2.67	0.374	8.88±3.14	8.23±2.61	0.103
TAPSE (mm)	15.00±4.84	17.38±5.21	0.154	16.31±5.36	15.88±5.36	0.215	16.36±7.02	14.95±5.94	0.062
LA area (cm^2)	23.13±6.64	22.88±4.16	0.845	27.12±7.15	26.57±6.12	0.168	27.91±7.61	27.24±7.74	0.298
RA area (cm^2)	20.00±6.74	19.50±3.96	0.775	22.78±6.36	22.49±5.50	0.482	23.70±6.08	23.93±7.71	0.775
T2 (ms)	52.00±2.39	48.88±2.90	0.002	52.08±3.04	52.45±3.30	0.084	52.23±2.71	55.47±3.99	<0.001
ECV*	0.46±0.05	0.40±0.06	0.009	0.47±0.08	0.49±0.09	<0.001	0.49±0.08	0.57±0.09	<0.001
ECV response*	Regression = 4 (50.0%)<br>Stable = 3 (37.5%)<br>Progression = 0 (0.0%)<br>Non-contrast CMR = 1 (12.5%)			Regression = 3 (2.3%)<br>Stable = 94 (72.3%)<br>Progression = 21 (16.2%)<br>Non-contrast CMR = 12 (9.2%)			Regression = 0 (0.0%)<br>Stable = 11 (24.4%)<br>Progression = 28 (62.2%)<br>Non-contrast CMR = 6 (13.3%)		

eTable 4. Changes in serum biomarkers, echocardiographic and cardiac magnetic resonance findings in patients with cardiac AL amyloidosis according to changes in native-T1 6-months after the initiation of chemotherapy. Reduced native-T1 was defined as a native-T1 reduction ≥50ms, stable native-T1 was defined as a change in native-T1 <50ms, and increased native-T1 was defined as a native-T1 reduction ≥50ms. *ECV measurements were available for the 164 patients who received gadolinium contrast during both their baseline and 6-month follow up CMR scans.

NT-proBNP = N-terminal pro-brain natriuretic peptide, IVSd = Interventricular septal diameter in diastole, RWT = Relative wall thickness, LS = Longitudinal strain, MWT = Maximal wall thickness, LV = Left ventricular, LVEF = Left ventricular ejection fraction, MAPSE = Mitral annular plane systolic excursion, TAPSE = Tricuspid annular plane systolic excursion, LA = Left atrial, RA = Right atrial, ECV = Extracellular volume.

© 2023 Ioannou A et al. *JAMA Cardiololgy.*

Baseline characteristics				
	**Reduced native-T1 (n = 24, 15.0%)**	**Stable native-T1 (n = 112, 70.0%)**	**Increased native-T1 (n = 24, 15.0%)**	**P-value**
**Demographics**				
Age (years)	59.26±11.58	64.73±11.35	63.88±8.34	0.115
Sex (male)	13 (54.2%)	65 (58.0%)	16 (66.7%)	0.653
**Serum biomarkers**				
NT-proBNP (ng/L)	2638 (914-5767)	2270 (927-4314)	1622 (553-5487)	0.750
Troponin (ng/L)	37 (28-70)	49 (30-93)	57 (26-122)	0.492
**Echocardiographic parameters**				
IVSd (mm)	13.42±2.98	14.06±2.54	14.50±2.65	0.403
RWT	0.66±0.19	0.68±0.17	0.70±0.18	0.669
E/e'	14.93±9.97	15.94±7.09	14.18±4.27	0.667
LS (%)	-14.76±3.98	-13.67±5.07	-14.31±4.46	0.455
**Cardiac magnetic resonance parameters**				
MWT (mm)	14.83±3.60	15.56±4.16	15.41±3.80	0.864
LV mass indexed (g/m^2)	88.83±32.59	98.38±35.73	97.63±33.35	0.416
LVEF (%)	67.67±12.00	64.50±10.51	65.79±11.41	0.190
MAPSE (mm)	8.64±3.14	8.43±3.14	7.91±3.10	0.636
TAPSE (mm)	17.48±5.75	15.75±5.70	15.55±5.07	0.404
LA area (cm^2)	20.75±4.85	26.62±7.70	27.04±7.32	0.450
RA area (cm^2)	20.75±4.67	22.95±7.16	21.83±6.09	0.400
Native T1 (ms)	1183.38±39.90	1157.12±61.50	1152.38±51.19	0.099
T2 (ms)	52.42±2.84	51.56±2.99	52.52±2.69	0.100
ECV*	0.47±0.07	0.47+0.09	0.48±0.09	0.933

eTable 5. Baseline demographics, serum biomarker, echocardiographic and cardiac magnetic resonance findings at diagnosis in patients with cardiac AL amyloidosis according to changes in native-T1 12-months after the initiation of chemotherapy. Reduced native-T1 was defined as a native-T1 reduction ≥50ms, stable native-T1 was defined as a change in native-T1 <50ms, and increased native-T1 was defined as a native-T1 reduction ≥50ms. *ECV measurements were only available for the 146 patients who received gadolinium contrast during their baseline and 12-month follow up CMR scans.

NT-proBNP = N-terminal pro-brain natriuretic peptide, IVSd = Interventricular septal diameter in diastole, RWT = Relative wall thickness, LS = Longitudinal strain, MWT = Maximal wall thickness, LV = Left ventricular, LVEF = Left ventricular ejection fraction, MAPSE = Mitral annular plane systolic excursion, TAPSE = Tricuspid annular plane systolic excursion, LA = Left atrial, RA = Right atrial, ECV = Extracellular volume.

© 2023 Ioannou A et al. *JAMA Cardiololgy.*

	Reduced native-T1 (n=24, 15.0%)			Stable native-T1 (n=112, 70.0%)			Increased native-T1 (n=24, 15.0%)		
	**Baseline**	**12 months**	**P-value**	**Baseline**	**12 months**	**P-value**	**Baseline**	**12 months**	**P-value**
**Serum biomarkers**									
Haematological response	CR = 18 (75.0%)<br>VGPR = 6 (25.0%)<br>PR = 0 (0.0%)<br>NR = 0 (0.0%)			CR = 51 (45.5%)<br>VGPR = 40 (35.7%)<br>PR = 14 (12.5%)<br>NR = 7 (6.3%)			CR = 4 (16.7%)<br>VGPR = 3 (12.5%)<br>PR = 13 (54.2%)<br>NR = 4 (16.7%)		
NT-proBNP (ng/L)	2638 (913-5767)	423 (128-1777)	<0.001	2270 (927-4314)	1568 (561-3475)	0.009	1622 (554-5487)	3150 (1161-8745)	0.007
NT-proBNP response	Improvement = 19 (79.1%)<br>Stable = 3 (12.5%)<br>Worse = 2 (8.3%)<br>Missing data = 0 (0.0%)			Improvement = 42 (37.5%)<br>Stable = 33 (29.5%)<br>Worse = 31 (27.7%)<br>Missing data = 6 (5.4%)			Improvement = 3 (12.5%)<br>Stable = 9 (37.5%)<br>Worse = 12 (50.0%)<br>Missing data = 0 (0.0%)		
**Echocardiographic parameters**									
IVSd (mm)	13.42±2.98	13.33±3.02	0.714	14.13±2.48	14.18±2.48	0.603	14.45±2.30	15.05±2.40	0.050
RWT	0.66±0.19	0.65±0.17	0.759	0.69±0.17	0.67±0.15	0.080	0.69±0.15	0.74±0.14	0.045
E/e’	14.93±6.84	12.04±5.24	0.007	16.26±7.24	15.77±6.52	0.416	14.39±4.28	16.71±6.25	0.064
LS (%)	-14.76±3.98	-16.68±4.00	0.004	-13.19±4.79	-13.09±4.73	0.754	-14.19±4.34	-13.50±4.55	0.274
**Cardiac magnetic resonance parameters**									
MWT (mm)	14.83±3.60	13.61±3.88	0.009	15.62±4.14	15.42±3.97	0.351	15.41±3.80	15.55±3.61	0.731
LV mass indexed (g/m^2)	88.83±32.59	85.29±36.54	0.505	98.59±35.82	99.27±42.99	0.798	97.63±33.35	104.54±37.67	0.070
LVEF (%)	67.67±12.00	67.21±8.45	0.801	63.77±10.51	62.92±11.03	0.309	65.79±11.41	61.50±12.40	0.009
MAPSE (mm)	8.71±3.20	9.38±3.28	0.095	8.48±3.14	8.34±2.55	0.581	7.91±3.10	7.55±1.82	0.515
TAPSE (mm)	17.39±6.15	19.67±5.21	0.093	15.67±5.68	15.65±5.51	0.966	15.55±5.07	15.05±4.97	0.413
LA area (cm^2)	24.38±4.85	22.88±4.85	0.066	26.62±7.70	26.09±5.77	0.220	27.04±7.32	27.83±5.65	0.407
RA area (cm^2)	20.70±4.73	20.17±4.08	0.556	22.95±7.16	22.96±5.92	0.970	21.83±6.09	23.79±7.47	0.107
T2 (ms)	52.33±2.85	49.42±2.00	<0.001	51.61±2.96	52.06±2.77	0.069	52.52±2.67	55.26±4.23	<0.001
ECV*	0.47±0.07	0.42±0.08	<0.001	0.47±0.09	0.47±0.09	0.243	0.48±0.09	0.56±0.09	<0.001
ECV response*	Regression = 18 (75.0%)<br>Stable = 5 (20.8%)<br>Progression = 0 (0.0%)<br>Non-contrast CMR = 1 (4.2%)			Regression = 13 (11.6%)<br>Stable = 77 (68.8%)<br>Progression = 10 (8.9%)<br>Non-contrast CMR = 12 (10.7%)			Regression = 0 (0.0%)<br>Stable = 1 (4.2%)<br>Progression = 22 (91.7%)<br>Non-contrast CMR = 1 (4.2%)		

eTable 6. Changes in serum biomarkers, echocardiographic and cardiac magnetic resonance findings in patients with cardiac AL amyloidosis according to changes in native-T1 12-months after the initiation of chemotherapy. Reduced native-T1 was defined as a native-T1 reduction ≥50ms, stable native-T1 was defined as a change in native-T1 <50ms, and increased native-T1 was defined as a native-T1 reduction ≥50ms. *ECV measurements were available for the 146 patients who received gadolinium contrast during their baseline and 12-month follow up CMR scans.

NT-proBNP = N-terminal pro-brain natriuretic peptide, IVSd = Interventricular septal diameter in diastole, RWT = Relative wall thickness, LS = Longitudinal strain, MWT = Maximal wall thickness, LV = Left ventricular, LVEF = Left ventricular ejection fraction, MAPSE = Mitral annular plane systolic excursion, TAPSE = Tricuspid annular plane systolic excursion, LA = Left atrial, RA = Right atrial, ECV = Extracellular volume.

© 2023 Ioannou A et al. *JAMA Cardiololgy.*

	Univariable		Multivariable with native-T1 and without ECV		Multivariable with ECV and without native-T1	
	HR (95% CI)	P-value	HR (95% CI)	P-value	HR (95% CI)	P-value
**Haematological response**						
CR	Reference		Reference		Reference	
VGPR	1.95 (0.73-5.21)	0.180	1.64 (0.61-4.42)	0.329	1.09 (0.37-3.29)	0.874
PR	6.98 (3.09-15.78)	<0.001	4.66 (1.96-11.11)	<0.001	2.77 (1.12-6.88)	0.028
NR	16.94 (7.38-38.89)	<0.001	11.32 (4.69-27.28)	<0.001	9.92 (4.12-23.90)	<0.001
**NT-proBNP response**						
Improvement	Reference		Reference		Reference	
Stable	0.90 (0.40-2.02)	0.804	0.98 (0.43-2.26)	0.961	1.19 (0.49-2.91)	0.699
Worsening	2.19 (1.10-4.36)	0.025	1.14 (0.56-2.32)	0.716	1.14 (0.54-2.41)	0.730
**Native T1 response**						
Reduced/stable	Reference		Reference		Reference	
Increased	3.92 (2.31-6.66)	<0.001	2.41 (1.36-4.27)	0.003	-	-
**ECV response**						
Regression/stable	Reference		Reference		Reference	
Progression	6.32 (3.47-11.50)	<0.001	-	-	4.67 (2.41-9.06)	<0.001
Harrell's c	-	-	0.799 (0.744-0.853)	<0.001	0.824 (0.769-0.879)	<0.001
AIC	-	-	477.24		385.68	

eTable 7. Univariable and multivariable analysis of mortality risk 6-months after the initiation of chemotherapy. Reduced native-T1 was defined as a native-T1 reduction ≥50ms, stable native-T1 was defined as a change in native-T1 <50ms, and increased native-T1 was defined as a native-T1 reduction ≥50ms. CR = Complete response, VGPR = Very good partial response, PR = Partial response, NR = No response, NT-proBNP = N-terminal pro-brain natriuretic peptide, ECV = Extracellular volume, AIC = Akaike information criterion.

© 2023 Ioannou A et al. *JAMA Cardiololgy.*
