# Supplementary material for: Tracking Treatment Response in Cardiac Light-Chain Amyloidosis With Native T1 Mapping
Source: JAMA Cardiol. 2023 Jul 19;8(9):848–52. doi: 10.1001/jamacardio.2023.2010 (PMC10357357; doi:10.1001/jamacardio.2023.2010)
Supplement: Supplement 2. — Data sharing statement [file jamacardiol-e232010-s002.pdf]

Data Sharing Statement

Ioannou. Tracking Treatment Response in Cardiac Light-Chain Amyloidosis With Native T1 Mapping. *JAMA Cardiol*. Published July 19, 2023. doi:10.1001/jamacardio.2023.2010

Data
Data available: No
